# Association between physical work demands and work ability in workers with musculoskeletal pain: cross-sectional study

**DOI:** 10.1186/s12891-020-03191-8

**Published:** 2020-03-14

**Authors:** Sebastian Venge Skovlund, Rúni Bláfoss, Emil Sundstrup, Lars L. Andersen

**Affiliations:** 1 Musculoskeletal Disorders and Physical Workload, National Research Centre for the Working Environment, Lersø Parkallé 105, 2100 Copenhagen, Denmark; 2 Department of Sports Science and Clinical Biomechanics, Research Unit for Muscle Physiology and Biomechanics, University of Southern Denmark, Odense, Denmark; 3 Sport Science, Department of Health Science and Technology, Aalborg University, Aalborg, Denmark

**Keywords:** Musculoskeletal disease, Occupational medicine, Ergonomics, Work ability, Physical work, Workplace, Sustainable employment

## Abstract

**Background:**

Musculoskeletal pain is common in the working population and may affect the work ability, especially among those with high physical work demands. This study investigated the association between physical work demands and work ability in workers with musculoskeletal pain.

**Methods:**

Workers with physically demanding jobs (*n* = 5377) participated in the Danish Work Environment Cohort Study in 2010. Associations between physical work ability and various physical work demands were modeled using cumulative logistic regression analyses while controlling for possible confounders.

**Results:**

In the fully adjusted model, bending and twisting/turning of the back more than a quarter of the workday (reference: less than a quarter of the workday) was associated with higher odds of lower work ability in workers with low-back pain (OR: 1.38, 95% CI: 1.09–1.74) and neck-shoulder pain (OR: 1.29, 95% CI: 1.01–1.64). When adding up the different types of demands, being exposed to two or more physical work demands for more than a quarter of the workday was consistently associated with lower work ability.

**Conclusions:**

Work that involves high demands of the lower back seems especially problematic in relation to work ability among physical workers with musculoskeletal pain. Regardless of the specific type of physical work demand, being exposed to *multiple* physical work demands for more than a quarter of the workday was also associated with lower work ability.

## Background

Musculoskeletal disorders (MSD) are widespread globally [1] and may profoundly affect the ability to work [2–4]. While MSD may affect all body regions, pain in the lower back or the neck is particularly prevalent and thereby constitute important causes of disability globally [1]. According to the 2018 round of the Danish Work Environment and Health Study, MSD and low work ability are more prevalent among Danish workers in physically demanding occupations than among the general working population [5]. Importantly, the literature indicates that these problems may be work-related, with occupational-related physical activity adversely impacting the involved parts of the body [6].

Work ability is a multifactorial construct that reflects the balance between occupational demands and the individual worker’s capacity [7]. Lower work ability has been associated with increased risks of work productivity loss [8], sickness absence [9–11] as well as premature exit from the labor market [10, 12] and ultimately all-cause mortality [13].

Multiple occupational and lifestyle factors may negatively influence work ability [14], including older age [15], obesity [16], mental and physical health problems [17, 18] as well as low physical capacity [14] and low levels of leisure-time physical activity [4, 19]. Furthermore, physical work – typically involving repetitive work, awkward postures and heavy lifting – has been associated with increased risk of MSD [6, 20, 21] and reduced work ability [14, 15, 22].

However, many workers with MSD are still able to work without the symptoms compromising work ability [22, 23]. It is plausible that working with MSD is easier when the physical work demands are low. Thus, exposure to a multitude of different physical work demands along with dose of exposure could be important factors related to the practical consequences of musculoskeletal disorders. Therefore, knowledge of the possible association between work ability and the exposure time and type, and the number of specific physical work demands would give practitioners at the workplace (both workers and managers) a larger knowledgebase to act on in the pursuit of preserving work ability among workers with MSD undertaking manual labor.

Thus, the aim of this cross-sectional study is to determine the association between work ability and physical work demands – both specific and combined demands – among physical workers with MSD in the upper body. We hypothesized that an exposure-response association would exist between an increased number of combined physical work demands and lower work ability.

## Methods

### Study design

The present cross-sectional study employ self-reported data on work ability and physical work demands from the 2010 round of the Danish Work Environment Cohort Study (DWECS). DWECS is a comprehensive questionnaire survey assessing work environment and health among the general working population residing in Denmark [4, 19]. Specific questions used in this study are specified below. The reporting of the study follows the guidelines for the reporting of observational studies in epidemiology (STROBE) [24].

### Ethics

This study has been reported to and registered by the Danish Data Protection Agency (journal number 2015-57-0074). Due to the Danish law, de-identified and anonymized data in questionnaires and register-based studies can be used for research without approval by ethical and scientific committees, and without obtaining informed consents.

### Participants

By September–October 2010, an invitation and the accompanied hard copy survey was sent to a about 20.000 Danish wage earners aged 18–59 years, which had been randomly drawn from the Central Population Register of Denmark. Those not responding received one or more reminders. A total of 53% (10,605 workers) responded to the questionnaire [4, 19]. The present study included only physical workers (*n* = 5377), identified by the following question: ‘How will you describe your physical activity level in your main profession?’. The respondents were classified as physical workers if they replied positively to one of the following response options: (i) ‘Mostly standing and walking work that otherwise is not physically demanding’, (ii) ‘Standing or walking work with some lifting- and bearing tasks’, (iii) ‘Heavy or fast work that is physically demanding’. Participants were excluded from the analyses if they replied ‘Mostly sedentary work that is not physically demanding’.

Not all participants filled in all survey questions for which reason the exact number of participants for each analysis varies.

### Explanatory variables

#### Physical work demands

The respondents answered the following questions in order to determine the exposure time to eight specific physical work demands: ‘Does your work cause you to: (i) stand in the same place, (ii) work with your back strongly bent forward without hand- and arm support, (iii) twist and bend your back several times per hour, (iv) have your arms raised to or above shoulder height, (v) perform repetitive arm movement/the same arm movements several times per minute (e.g. package work, mounting, machine feeding, carving), (vi) squat or kneel, (vii) push or pull (viii) carry or lift?’

In the analyses, questions two and three as well as questions seven and eight were collapsed in order to obtain more statistical power and because a previous study have found them to be highly correlated (spearman’s r = 0.60–0.61, see), and thus basically expressing the same exposure. The response options to all questions were: ‘1) Almost all the time, 2) Approximately 3/4 of the time, 3) Approximately 1/2 of the time, 4) Approximately 1/4 of the time, 5) Rarely/very little, or 6) Never’ [25, 26]. The response categories were defined as 100, 75, 50, 25, 12½ and 0% of the duration of a total workday, respectively. Based on previously reported associations between physical work demands and post-work bodily fatigue [27] and sickness absence [25, 28], 25% of the workday was selected as cut-point for being exposed to all physical work demands, except for ‘standing in the same place’ where the cut-point was 50% of the workday.

#### Musculoskeletal pain

Participants were classified as having musculoskeletal pain if they reported average pain intensities during the past 3 months in the low-back (***LBP***), neck-shoulder (***NSP***), and/or arm (including hands, forearm, and elbow, ***AP***) to be ≥4 on a scale from 0 to 9, where 0 is no pain and 9 is worst possible pain [29, 30].

### Outcome variable

#### Work ability

The work ability of the respondents was assessed by the following single-item question from the work ability index (WAI) [7]: ‘How do you rate your current work ability with respect to the physical demands of your work?’ with the following response options: 1) ‘Excellent’, 2) ‘Very good’, 3) ‘Good’, 4) ‘Fair’, or 5) ‘Poor’. Subsequently, response options were converted to a scale from 0 to 100, where a score of 0 corresponded to poor, a score of 25 corresponded to fair, 50 corresponded to good, 75 corresponded to very good, and a score of 100 corresponded to excellent work ability [4, 19].

### Control variables

We controlled for the following potential covariates: age (years, continuous), gender (categorical; ‘male’ or ‘female’), smoking status (categorical; ‘No, never’, ‘Ex-smoker’ and ‘Yes’), body mass index (BMI) (continuous; kg/m^2^), musculoskeletal pain (continuous scale 0–9, only 4–9 included in the analyses; see definition above), psychosocial work environment and chronic disease (see definitions below).

Chronic disease was assessed by the question: ‘Has a doctor ever told you that you have or have had one or more of the following diseases?’: ‘depression’, ‘asthma’, ‘diabetes (all types)’, ‘cardiovascular disease’, and ‘cancer’, with response options being ‘Yes’ and ‘No, never’.

Psychosocial work environment was assessed on a continuous scale from 0 to 100 on questions regarding emotional demands and influence at work derived from the Copenhagen Psychosocial Questionnaire [31].

We controlled for these variables because previous studies have shown associations between work ability and these occupational and lifestyle factors [14], i.e., age [15, 22], gender [15, 22], smoking [15], overweight [16], MSD [2–4], psychosocial work environment [10, 22] and chronic disease [17, 18].

### Statistical analyses

The statistical analyses were performed in the SAS statistical software for Windows (Proc Logistic, SAS version 9.4, SAS Institute, Cary, NC) and step-wise controlled for potential confounders. Statistical model 1 was adjusted for age and gender, whereas the fully adjusted model 2 was additionally adjusted for smoking status, body mass index (BMI), psychosocial work environment, MSD and chronic disease. Using cumulative logistic regression analyses, we estimated associations between work ability (outcome variable) and specific physical work demands (exposure variables) in separate groups of workers with pain (≥4) at three different body sites (neck/shoulder, low-back and arm). We did not impute missing data. Results are reported as ORs and 95% confidence intervals (CI) unless otherwise stated.

## Results

Table 1 illustrates the baseline characteristics of the study sample.

**Table 1:** Demographics and lifestyle characteristics of the included physical workers

	*n*	Mean	SD	%
Age (years)	5377	43.0	12.2	
Gender				
Men	2451			45.6
Women	2926			54.4
BMI				
Underweight	46			0.9
Normal	2665			51.2
Overweight	1767			33.9
Obese	729			14.0
Smoking				
No, never	2374			45.3
Ex-smoker	1456			27.8
Yes	1415			27.0
Diabetes				
No, never	5099			96.9
Yes	165			3.1
Cardiovascular disease				
No, never	5026			95.5
Yes	235			4.5
Cancer				
No, never	5080			96.6
Yes	181			3.4
Depression				
No, never	4581			87.0
Yes	683			13.0
Physical activity at work				
Mostly standing and walking work that otherwise is not physically demanding	2425			45.1
Standing or walking work with some lifting- and bearing tasks	2456			45.7
Heavy or fast work that is physically demanding	496			9.2
Work ability				
Excellent	1295			24.7
Very good	2054			39.1
Good	1410			26.9
Fair	407			7.8
Poor	86			1.6

*BMI* body mass index (kg/m^2^)

### Physical work demands and work ability

Table 2 shows associations between physical work demands and work ability. After adjusting for age and gender, working more (as compared to less) than 25% of the workday with the back strongly bent or twisted/turned was significantly associated with lower work ability in workers with low-back pain (OR: 1.47, 95% CI: 1.18–1.83) and neck-shoulder pain (OR: 1.42, 95% CI: 1.12–1.79). Likewise, repetitive arm work significantly associated with lower work ability in workers with low-back pain (OR: 1.26, 95% CI: 1.02–1.56) and neck-shoulder pain (OR: 1.38 95% CI: 1.10–1.72. In the fully adjusted model, bending or twisting/turning of the back for more than a quarter of the workday was associated with lower work ability in workers with low-back (OR: 1.38, 95% CI: 1.09–1.74) and neck-shoulder pain (OR: 1.29, 95% CI: 1.01–1.64). No associations were found between any physical work demand and work ability in workers with arm pain, neither in the minimally nor the fully adjusted models.

**Table 2:** Odds ratios (OR) and 95% confidence intervals (95% CI) for Physical work demands and work ability among workers with region-specific MSD in physically demanding jobs

	Model 1	Model 2
	OR (95% CI)	OR (95% CI)
Low-back pain *n* = 1758		
1. Standing in the same place	1.05 (0.82–1.34)	0.97 (0.75–1.25)
2. Back strongly bent or frequent twisting/turning of the back	**1.47 (1.18–1.83)**	**1.38 (1.09–1.74)**
3. Arms at or above shoulder height	1.01 (0.82–1.25)	0.93 (0.75–1.16)
4. Repetitive arm movement	**1.26 (1.02–1.56)**	1.08 (0.86–1.35)
5. Squatting or kneeling	0.99 (0.80–1.23)	1.03 (0.82–1.28)
6. Pushing/pulling or lifting/carrying	1.19 (0.96–1.47)	1.01 (0.81–1.26)
Neck-shoulder pain *n* = 1635		
1. Standing in the same place	1.08 (0.84–1.39)	1.07 (0.82–1.39)
2. Back strongly bent or frequent twisting/turning of the back	**1.42 (1.12–1.79)**	**1.29 (1.01–1.64)**
3. Arms at or above shoulder height	1.15 (0.92–1.43)	1.05 (0.83–1.32)
4. Repetitive arm movement	**1.38 (1.10–1.72)**	1.02 (0.80–1.30)
5. Squatting or kneeling	1.01 (0.81–1.27)	1.07 (0.85–1.36)
6. Pushing/pulling or lifting/carrying	1.19 (0.95–1.48)	1.06 (0.84–1.34)
Hand-wrist pain *n* = 1025		
1. Standing in the same place	0.92 (0.68–1.26)	0.83 (0.60–1.16)
2. Back strongly bent or frequent twisting/turning of the back	1.28 (0.96–1.70)	1.12 (0.83–1.51)
3. Arms at or above shoulder height	1.05 (0.79–1.39)	0.95 (0.71–1.27)
4. Repetitive arm movement	1.25 (0.95–1.63)	1.14 (0.85–1.52)
5. Squatting or kneeling	1.15 (0.86–1.53)	1.21 (0.90–1.64)
6. Pushing/pulling or lifting/carrying	1.17 (0.89–1.55)	0.99 (0.74–1.34)

Model 1: Adjusted for age and gender

Model 2: model 1 + lifestyle (smoking, BMI), psychosocial work environment (influence at work, emotional demands), pain (intensity in the low-back, neck-shoulder, and hand-wrist), chronic disease (depression, diabetes, cardiovascular disease, cancer)

Significant differences (*P* < 0.05) from reference are marked in **bold**. Cut-points for physical work demands were set at 25% of workday, except ‘Standing in the same place’, which was set at 50% of workday. Estimates are given as OR and 95% CI

Table 3 reports the associations between the summed numbers of combined physical work demands more than a quarter of the workday and work ability. The data demonstrates a borderline significant (trend test *p* = 0.0525) exposure-response relationship between increased number of combined physical work demands and lower work ability. Still, while exposure to only one physical work demand for above a quarter of the workday was associated with reduced work ability in the minimally adjusted model (OR: 1.20, 95% CI: 1.03–1.40), this association disappeared in the fully adjusted model (OR: 1.03, 95% CI: 0.88–1.21). Compared to not being exposed to any physical work demand for a quarter of the workday, exposure to two or more of the physical work demands – regardless of the specific type – for more than a quarter of the workday associated with lower work ability in both models.

**Table 3:** Odds ratios (OR) and 95% confidence intervals (95% CI) for summed number of physical work demands and work ability

Number of exposures	*n*	%	Model 1	Model 2
			OR (95% CI)	OR (95% CI)
0	1342	25.4	1	1
1	1029	19.5	**1.20 (1.03–1.40)**	1.03 (0.88–1.21)
2	931	17.6	**1.81 (1.55–2.11)**	**1.29 (1.09–1.52)**
3	904	17.1	**2.01 (1.71–2.36)**	**1.28 (1.08–1.51)**
4 or more	1084	20.5	**2.78 (2.39–3.25)**	**1.45 (1.22–1.72)**

Model 1: Adjusted for age and gender.

Model 2: model 1 + lifestyle (smoking, BMI), psychosocial work environment (influence at work, emotional demands), pain (intensity in the lower back, neck-shoulder, and arm), chronic disease (depression, diabetes, cardiovascular disease, cancer).

Significant differences (*P* < 0.05) from reference are marked in **bold**. Cut-points for physical work demands were set at 25% of workday, except ‘Standing in the same place’, which was set at 50% of workday.

Overall, age did not significantly influence the association between number of physical work demands and work ability (*P* = 0.69).

## Discussion

Our study shows that work involving high demands of the lower back seems especially problematic in relation to work ability among physical workers with musculoskeletal pain in the upper body. Regardless of the specific type of work demand, being exposed to *multiple* physical work demands for more than a quarter of the workday was also associated with lower work ability.

### The relation between mechanical workloads and health

Spending more than a quarter of the working day with a bent and twisted back significantly increased the odds for having lower work ability in workers with pain in the lower back or neck-shoulder region. However, no significant associations were observed with the remaining physical work demands (i.e. standing in the same place, arms raised to or above shoulder height, repetitive arm movement, squatting or kneeling, pushing or pulling, and lifting or carrying) and work ability.

Applying the same cut-off limits for exposure time as we did in this study, we have previously shown that spending more than a quarter of the total work time with a bent or twisted back increases the risk of long-term sickness absence (LTSA) among the general working population and blue-collar workers, respectively, even after adjusting for baseline age, gender, psychosocial work environment, lifestyle, musculoskeletal and mental disorders, and socioeconomic status [25]. Likewise, bending and/or twisting the back more than 25% of the workday has been associated with increased whole-body fatigue among physical workers [27], which in itself may increase the risk of LTSA [32]. In an age- and gender-controlled model among the general working population in Norway, Sterud et al. also identified an association between LTSA and spending a quarter of the working hours with the upper body forwardly bent (or with awkward lifting), but this association disappeared after controlling for additional relevant confounders [28].

Repetitive arm work has also been associated with an increased risk of LTSA among blue-collar workers [25] and with whole-body fatigue in physical workers below 50 years of age [27]. A significant association to LTSA was only apparent in workers doing repetitive arm movement for at least three quarters of their workday, however, and thus not in workers doing repetitive arm work for only a quarter of their workday [28].

Even though we did not observe a significant exposure-response relationship between increased number of combined physical work demands and lower work ability, being exposed to two or more of the physical work demands for more than a quarter of the workday associated consistently with lower work ability compared to not being exposed to any of the specific physical work demands.

Consistent with our results, exposure to a higher *number* of physical work demands – regardless of the type – has previously been associated with higher risks of whole-body fatigue [27] and LTSA [25, 28], which may indicate an importance of reducing the total workload in prevention of work-related ill health.

#### Workplace-based exercise and tailored work tasks

Since physical work ability represents the balance between physical work demands and individual physical capacity, both reducing physical work demands and increasing capacity by the use of workplace exercise could potentially be a powerful workplace strategy for workers with MSD. The literature indicates that physical training performed during the workday can be effective in terms of preventing and treating MSD [6]. Although the meta-analytic evidence suggests a small positive effect on work ability when pooling studies of individually focused workplace interventions, the vast majority of interventions have failed to improve work ability [33], underlining the multifactorial nature of the work ability concept. While some workplace-based strength training interventions have diminished pain and prevented deterioration of work ability in physical workers [34, 35], others have reported decreased pain without any effects on work ability [36, 37]. Thus, although more high quality studies are warranted, appropriately frequent and intensive strength training at the workplace may be a viable strategy to increase or at least prevent deterioration of work ability.

Tailoring the work to the capacities and age of the worker may also serve as a means to preserve work ability and thus prevent disability pension and thereby create sustainable employment [38], especially concerning the high number of older workers with MSD. In contrast to Danish survey data [5], recent Nordic studies using objective measurements have suggested that older physical workers experience similar or even higher occupational physical demands compared to younger workers [38, 39], underlining a relevant room for improvement.

#### Strengths and limitations

The present study has several strengths and limitations. First, our results may be biased by the fact that we are only considering physical workers that are still capable of working (healthy worker effect), and not those that have left the labor market prematurely due to ill health.

Second, it is important to distinguish between the applied statistical models. Overall, the associations are stronger in model 1 than in model 2, and it is possible that model 1 overestimates the associations, while model 2 may underestimates the associations.

Third, the present cross-sectional design does not allow causal inferences to be made, i.e. high physical work demands may lead to lower work ability and vice versa.

Fourth, the self-rated assessment of physical work demands may be biased and less accurate than if objective measurements (i.e. 3D motion analysis or accelerometry) had been applied [40], and accordingly, overestimation of the time and levels of exposure to the specific work demands loads may constitute a risk of bias. Additionally, a potential exposure-response relationship between the duration of physical work demands and work ability may be overlooked using the specific 25% cut-off. For instance, it is possible that exposure to a single physical work demand for three quarters or more of the workday may be more detrimental to the work ability than being exposed to several physical work demands to a lower extent daily. Furthermore, this study only investigated pain in the upper body and low-back, and these findings may not apply to pain in lower body regions.

Our study methodology contains several strengths as well. First, the representative and large sample strengthens the statistical power and thereby reduces the chances of statistical type II errors. In addition, the adjustment for various relevant covariates is also a strength of the study. We used the particular single-item work ability assessment (originating from the WAI) because of its simplicity and ease of use and because previous studies have reported it to be a valid and reliable alternative to the full and more resource demanding WAI [15, 41]. Furthermore, given that MSD and low work ability are more prevalent among physical workers, the fact that we assessed *physical* work ability in relation to *physical* work demands may provide particularly useful knowledge for workplaces and industries characterized by high physical work demands. Also, we studied the summed number of physical work demands, which likely reflects the total accumulated physical strain during work better than only examining single work demands.

## Conclusions

Work that involves high demands of the lower back may be especially problematic in relation to work ability among physical workers with musculoskeletal pain. Regardless of the specific type of physical work demand, exposure to multiple physical work demands for more than a quarter of the workday associated with lower work ability. These findings could provide useful knowledge for employers and employees in physical trades in order to maintain work ability of physical workers with pain in the low-back or neck-shoulder.

## Data Availability

The datasets used and/or analyzed during the current study will be available from Professor Lars L. Andersen on a reasonable request.

## Abbreviations

95% CI: 95% confidence intervals
BMI: Body mass index
DWECS: The Danish Work Environment Cohort Study
OR: Odds ratio
MSD: Musculoskeletal pain
WAI: Work ability index
LBP: Low-back pain
NSP: Neck-shoulder pain
AP: Pain in arms (including hands, forearm, and elbow)
LTSA: Long-term sickness absence
